# A Stable Finite-Difference Scheme for Population Growth and Diffusion on a Map

**DOI:** 10.1371/journal.pone.0167514

**Published:** 2017-01-13

**Authors:** W. P. Petersen, S. Callegari, G. R. Lake, N. Tkachenko, J. D. Weissmann, Ch. P. E. Zollikofer

**Affiliations:** 1 Anthropological Institute and Museum, Univ. of Zürich, Zürich, Switzerland; 2 Seminar for Applied Mathematics, ETH, Zürich, Switzerland; 3 Institute for Computational Science, Univ. of Zürich, Zürich, Switzerland; Universita degli Studi di Catania, ITALY

## Abstract

We describe a general Godunov-type splitting for numerical simulations of the Fisher–Kolmogorov–Petrovski–Piskunov growth and diffusion equation on a world map with Neumann boundary conditions. The procedure is semi-implicit, hence quite stable. Our principal application for this solver is modeling human population dispersal over geographical maps with changing paleovegetation and paleoclimate in the late Pleistocene. As a proxy for carrying capacity we use Net Primary Productivity (NPP) to predict times for human arrival in the Americas.

## Introduction

There is considerable interest in modeling population dynamics at large spatial and temporal scales, for example the modern human out-of-Africa dispersal [1–4] or Neanderthal dispersal and extinction [5]. These models are required to interpret local and global patterns of genetic, phenetic and cultural variation [1, 6–9].

Fisher [10] studied the problem—via a growth-diffusion equation—of an analogous but one-dimensional situation: the propagation of an advantageous genetic mutation within an already-present population, situated along a coast line. Kolmogorov, Petrovskii and Piskunov [11] were more general; in particular, their analysis treated the two-dimensional case. Such a model (called Fisher/KPP in the following) was applied to the dispersal and growth of a population by Skellam [12], and serves as an important control for designing and validating other more complex spatiotemporal population models [5]. Coupling population dynamics with models of large-scale changes in continental topography, climate, and ecosystem productivity is necessary to understand the role of environmental constraints on patterns of genetic, phenetic, and cultural variation among human populations [5].

Here we present a stable and efficient finite-difference solver for the Fisher/KPP equation on the 2-D domains of geographical maps, and show how it can be extended to include environmental fluctuations. In a brief outline of our paper, we will: review the derivation of the Fisher/KPP equation (Section **The Fisher/KPP equation**); develop finite-difference schemes in 1 and 2 dimensions for variable environmental carrying capacity K (Section **Numerical methods and splitting**) depending on both space and time K(x,t) (Section **Space—time dependent capacity maps**); and show our application of this technique to the out-of-Africa dispersal of *Homo sapiens* by using net primal productivity (NPP) [1] as a proxy for K (Section **World-wide hominin dispersal**).

## The Fisher/KPP equation

An intuitive way to get the Fisher/KPP equation [10–12] is as follows. A current **j** of particles (e.g., individuals) moving across an interface located at **x** is proportional to the gradient of the population density *p* (called “Fickian diffusion”)

$$\begin{array}{c} j=-c\;\nabla p. \end{array}$$

The rate of change of *p* is then given by the mass balance equation [13], which for Fickian diffusion reads

$$\begin{array}{c} \frac{\partial p}{\partial t}-c\;\nabla^{2}p=\rho . \end{array}$$

If *ρ* = 0, this is the heat equation when *c* = *D*/2 and *D* is the diffusion coefficient. For lack of a better model, we assume *c* is a constant (Young & Bettinger [4]). In Subsection **Parameter optimization**, we optimize on *D* = 2*c*. The source term *ρ* is usually modeled by a logistic growth function, ρ=λp(1-p/K), and gives the Fisher/KPP equation

$$\begin{array}{c} \frac{\partial p}{\partial t}=c\;\nabla^{2}p+\lambda \left(1-\frac{p}{K}\right)p, \end{array}$$ (1)

where K is called *carrying capacity* and *λ* is the growth rate. In Eq (1) we wrote K as a constant. Since *ρ* is a local density, it can be space, **x**, and time, *t*, dependent. To get relative density information, all we need is that there is an *upper limit* $\bar{K}$, in which case $0\leq p\leq \bar{K}$. If K(x,t) is evolving, locally the solution $p\leq K\left(x,t\right)\leq \bar{K}$. As in Table 1, $\bar{K}$ can be scaled to unity, although obviously only the relative density $p/\bar{K}$ can be computed. A scaled version for constant K is given in Eq (2), while space and space-time dependent K(x,t) versions are given in the Section **Space-time dependent capacity maps**. Fluctuations in climate produce environmental changes in vegetation, sea levels, opening/closing of land bridges, waxing/waning of ice sheets, and perturbations to habitable areas in general. Thus, time-dependent environments compel us to deal with space- and time-dependent K(x,t) (see Subsection **Time interpolation of maps**), Section **Space-time dependent capacity maps**, Eq (9). Fisher and KPP were particularly interested in the traveling wave case, *p*(**x**, *t*) = *f*(**x**_0_ + **v***t*). It was shown in [14, 15] that asymptotically (large *t*), the speed is $v=2\sqrt{\lambda c}=\sqrt{2rD}$ in our notation. By the rescalings show in Table 1, for constant K the Fisher/KPP Eq (1) is written

$$\begin{array}{c} \frac{\partial u}{\partial t}=\frac{1}{2}\;\nabla^{2}u+\left(1-u\right)u, \end{array}$$ (2)

where the only sensible solutions have 0 ≤ *u* ≤ 1. The initial distribution *u*(**x**, 0) = *u*_0_(**x**) must be defined for all **x** on the habitable regions of the map.

**Table 1:** Variables in left column are scaled versions of those in right column.

In Eq (2)	In Eq (1)
x	$\sqrt{\frac{\lambda }{2c}}\;x$
*t*	*λt*
*u*	p/K

In Murray ([14], eq. (11.17)) our growth coefficient *λ* is called *r* and *c* is denoted by *D*, whereas in Young and Bettinger [4] the growth coefficient is *R* and the diffusion coefficient is *K*. We use *r* to denote radius in Section **Numerical methods and splitting**, and *k* for the CFL parameter, so *λ* and *D* are used here and that next section. Elsewhere, *λ* = *r* is used interchangeably. These inputs to our code are given in units of yr^−1^ and km^2^/yr, respectively.

## Numerical methods and splitting

In one dimension, Eq (2) can be solved using the MatLab function pdepe, or if the system is two-dimensional but rotationally symmetric, pdepe can again be used with the radial part of the Laplace operator in cylindrical coordinates,

$$\begin{array}{c} \nabla^{2}=\frac{1}{r}\frac{\partial }{\partial r}r\frac{\partial }{\partial r}+\text{non-contributing}\;\text{terms}, \end{array}$$

requiring only that one sets a pdepe parameter m=1. What is important is that because MatLab function pdepe is robust, it is a valuable verification tool for testing our more general solver when comparisons can be made.

### The finite-difference scheme

Since the map on which we will be working is a pixelized plane, an obvious method uses finite differences. First, however, let us examine the 1-D case for Eq (2). In this situation, the second order derivative becomes a differencing operator in matrix form acting on the vector {*u*_*j*_, *j* = 1, *n*}, where *u*_*j*_ = *u*(*x*_0_ + (*j*−1)Δ*x*),

$$\begin{array}{c} \frac{\partial^{2}u}{\partial x^{2}}\to \frac{1}{{\left(\Delta x\right)}^{2}}Au, \end{array}$$

where the matrix *A* is

$$\begin{array}{c} A=\left(\begin{array}{cccccc} -2 & 1 & 0 & 0 & \ldots & 0\\ 1 & -2 & 1 & 0 & \ldots & 0\\ 0 & 1 & -2 & 1 & \ldots & \\ 0 & 0 & 1 & \ldots & & \\ 0 & 0 & \ldots & 0 & 1 & -2 \end{array}\right). \end{array}$$ (3)

If *h* is the time step, the Courant—Friedrichs—Lewy (CFL) parameter [16] is

$$\begin{array}{c} k=\frac{h}{2{\left(\Delta x\right)}^{2}}. \end{array}$$ (4)

An explicit integrator for Eq (2) would require *k* < 1/4 [16, 17]. In our case, because boundary conditions are so irregular on a map and there are strong spacial variations in K, we are uninterested in a method of higher order than second because stability is more important [18].

Using this notation, the lowest order approximation is Euler’s method which estimates the next step *u*(*t* + *h*) by

$$\begin{array}{c} u_{E}=u\left(t\right)+k\;Au\left(t\right)+h\;\left(1-u\left(t\right)\right)u\left(t\right), \end{array}$$ (5)

which should be considered a vector equation in *u*(*t*) = {*u*_*j*_(*t*), *j* = 1, *n*}. The logistic terms, which are diagonal, should be taken to mean ((1−*u*)*u*)_*j*_ = (1−*u*_*j*_)*u*_*j*_ for *j* = 1, 2, …, *n*. Euler’s method is both low-accuracy and generally unusable if it is used alone over too many steps. This method is in principle conditionally stable if the step size is forced to be small enough. A step size which is too small, however, actually drives errors up, not down: e.g., C. Wm. Gear’s Fig. 1.8 [19], page 19. Euler’s method is *O*(*h*) accurate so is useful as an explicit estimate (predictor) inside *O*(*h*) terms, and thus yields *O*(*h*^2^) accuracy for such terms. An application of the trapezoidal rule yields

$$\begin{array}{ccc} u(t+h) & = & u\left(t\right)+\frac{k}{2}\left(Au(t+h)+Au(t)\right)\\ & & \;+\frac{h}{2}\left((1-u(t+h))u(t+h)+(1-u(t))u(t)\right) \end{array}$$ (6)

and is an *O*(*h*^2^) + *O*((Δ*x*)^2^) accurate procedure. However, solving the quadratic vector Eq (6) for *u*(*t* + *h*) is awkward. To the same *O*(*h*^2^) accuracy, we use a semi-implicit procedure which uses the Euler estimate Eq (5) to modify one of the terms, (1−*u*(*t* + *h*)), in Eq (6):

$$\begin{array}{ccc} u(t+h) & = & u\left(t\right)+\frac{k}{2}\left(Au(t+h)+Au(t)\right)\\ & & \;+\frac{h}{2}\left(\left(1-u_{E}\right)u\left(t+h\right)+\left(1-u\left(t\right)\right)u\left(t\right)\right). \end{array}$$ (7)

Eq (7) can be solved as a linear system,

$$\begin{array}{c} \left(1-\frac{k}{2}A-\frac{h}{2}\left(1-u_{E}\right)\right)u\left(t+h\right)=u\left(t\right)+\frac{k}{2}Au\left(t\right)+\frac{h}{2}\left(1-u\left(t\right)\right)u\left(t\right), \end{array}$$ (8)

because the matrix, $1-\frac{k}{2}A-\frac{h}{2}\left(1-u_{E}\right)$, on the left hand side is explicit, as is the right hand side. That is, this matrix and the right hand side contain only old data, namely just information from the previous step, *u*(*t*). Euler estimate *u*_*E*_ Eq (5) is an explicit one step computation using old data *u*(*t*). Significant advantages are: the matrix on the left hand side is tridiagonal with constants on the sub/super-diagonals, and the diagonal terms are *O*(1) strong. The procedure Eq (8) is only linearly stable but we find empirically that it gives good results when compared to pdepe when comparisons to this MatLab function are appropriate.

**Fig 1:**
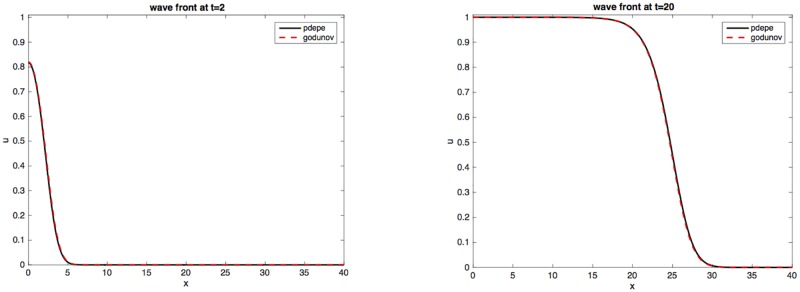
Godunov vs. pdepe. Left: Godunov vs. pdepe at *t* = 2. Right: same at time *t* = 20. Only *x* > 0 data are shown. The time step *h* = 1/12 and Δ*x* = 1/5. Initial data: *u*(*x*) = 1 if |*x*| < 1, zero otherwise.

Fig 1 shows the results for *h* = 1/12, Δ*x* = 1/5 compared to pdepe. Notice that at both *t* = 2 and *t* = 20 the agreement is quite satisfactory. The CFL number, *k* = 1, used to get Fig 1 is much larger than would be possible with an explicit method [17].

## Space-time dependent capacity maps

Fluctuations in climate produce environmental changes in vegetation, sea levels, opening/closing of land bridges, waxing/waning of ice sheets, and perturbations to habitable areas in general. Thus, changing environments compel us to develop a procedure Eq (9) for the case when K(x,t) depends on both space and time.

Since Eq (8) is basically the trapezoidal method (see section 5.3 in [17]), the modification for a space—time dependent K(x,t) is as follows:

$$\left(1-\frac{k}{4}A_{x}\right)u^{⋆}=\left(1+\frac{k}{4}A_{x}\right)u\left(t\right)$$ (9a)

$$u_{E}=u^{⋆}+kA_{y}u^{⋆}+h\left(1-\frac{u^{⋆}}{K\left(t\right)}\right)u^{⋆}$$ (9b)

$$\left(1-\frac{k}{2}A_{y}-\frac{h}{2}\left(1-\frac{u_{E}}{K\left(t+h\right)}\right)\right)u^{⋆⋆}=\left(1+\frac{k}{2}A_{y}+\frac{h}{2}\left(1-\frac{u^{⋆}}{K\left(t\right)}\right)\right)u^{⋆}$$ (9c)

$$\left(1-\frac{k}{4}A_{x}\right)u\left(t+h\right)=\left(1+\frac{k}{4}A_{x}\right)u^{⋆⋆},$$ (9d)

where we have suppressed the **x** dependence of K(x,t) for simplicity of notation. In Eq (9), the operators *A*_*x*_ and *A*_*y*_ are the same finite difference operators as in Eq (3) but for directions *x* and *y*, respectively. For simulations on a lattice, *u*_*ij*_(*t*) = *u*(*x*_0_ + (*i*−1)Δ*x*, *y*_0_ + (*j*−1)Δ*y*, *t*), where 1 ≤ *i* ≤ *N*_*x*_, 1 ≤ *j* ≤ *N*_*y*_ and Δ*x* = Δ*y*, the following gives the action of the *A*_*x*_, *A*_*y*_ operators:

$$\begin{array}{ccc} A_{x}u_{i,j} & = & u_{i-1,j}-2u_{i,j}+u_{i+1,j},\\ A_{y}u_{i,j} & = & u_{i,j-1}-2u_{i,j}+u_{i,j+1}. \end{array}$$

A compression scheme and code outline given in Appendix **Map segmentation** show that only a maximum of one row or column (i.e., max(*N*_*x*_, *N*_*y*_)) of storage is needed for *u*^⋆^ and *u*^⋆⋆^.

We use the simple regularization of each version of 0≤u≤K in Eq (9) at every **x**−point, namely

$$\begin{array}{cc} 0\leq u^{⋆} & \leq K(x,t),\\ 0\leq u_{E} & \leq K(x,t+h),\\ 0\leq u^{⋆⋆} & \leq K(x,t+h). \end{array}$$

For example: use Eq (9b) to compute *u*_*E*_, then set *u*_*E*_ ← max(0, *u*_*E*_), followed by $u_{E}\leftarrow \min\left(K\left(x,t+h\right),u_{E}\right)$. Intermediate values *u*^⋆^ and *u*^⋆⋆^ are constrained similarly.

### Fisher/KPP on geographical maps

In order to solve Fisher/KPP on a geographical map, one segments the map into *x*- and *y*-direction pieces, imposing boundary conditions at their endpoints; the solver acts on each segment independently, changing the directions *x* → *y* Eqs (9a) to (9b), then *y* → *x* Eqs (9b) to (9d) [17] of integration as in Eq (8). This approach also lends itself to efficient parallelization. In the **Map segmentation appendix** we illustrate the scheme with an illustrative MatLab code sample.

## Boundary conditions for Fisher/KPP

Two versions for boundary conditions were implemented and tested: (1) zero solution Dirichlet, and (2) zero net flux Neumann conditions. Both variations require some explanation.

### Dirichlet BCs

In this case, a border of zero pixels were added to the land masses. In our Godunov method, each direction (*X*, respectively *Y*) segments acquire two zero pixel end points, and the resulting *n*_*seg*_ + 2 one dimensional difference equations are solved, but only the *n*_*seg*_ land mass pixel values of the solution are updated. For example, in the Appendix **Map segmentation**, an *X* direction segment passing through equatorial central Africa has *n*_*seg*_ pixels, each of which also has a corresponding space/time dependent carrying capacity K(x,t). Two zero pixels are added, one at each end, but K(x,t) for those additional points are not relevant, nor used. Single pixel land mass segments thus become 3 pixels, where only the middle point is updated. The differencing operator (3) becomes very simple with three elements/row (1, -2, 1). Simulations with high resolution, say *N*_*x*_ = 720 and *N*_*y*_ = 360, show that the coastal region populations look too small. This is because of smoothing between zero (water) and the second adjacent pixel away. Our ancestors fished, so we concluded Dirichlet BCs seem inappropriate.

### Neumann BCs

In consequence, we use zero net flux, ∂*u*/∂*x* = 0 or ∂*u*/∂*y* = 0 into the sea conditions. This is more awkward and two issues must be dealt with:

- Derivative information (*u*_*x*_, *u*_*y*_ to *O*(Δ*x*^2^)) must be available, or approximated. From LeVeque [17], we know that when *n*_*seg*_ > 3, we may use $\partial u/\partial x\approx (\frac{3}{2}u\left(x\right)-2u\left(x+\Delta x\right)+\frac{1}{2}u\left(x+2\Delta x\right))/\Delta x$ as a one-sided (to the right, here) difference approximation. If *n*_*seg*_ ≤ 3, however, things are clumsier.
- Furthermore, because our Godunov splitting advances two one half steps of the heat equation per time step, there is the question of whether the solver simulates these half-steps in a properly posed way. It is well known (again [17]) that the heat equation with Neumann boundary conditions can be an ill-posed problem. This is easy to see: $u_{t}=\frac{1}{2}u_{xx}$ with *u*_*x*_ = 0 at the end points remains invariant to the shift *u* → *u* + *C*, for any constant *C*. Thus, in that case, the solution cannot be unique. Uniqueness must be imposed by the logistics term in (2). That term is not invariant under the shift, so uniqueness is assured. Only single half time—steps for the heat equation steps, Eqs (9a) and (9d), are taken.

If *n*_*seg*_ > 3, the resulting linear equations to be solved are of the form Eq (9c).

$$\begin{array}{cc} \left(\begin{array}{ccccc} 1, & -4/3, & 1/3 & \cdots & 0\\ -\frac{k}{2}, & 1+k-\frac{h}{2}\left(1-u_{E,2}\right), & -\frac{k}{2} & \cdots & 0\\ 0 & -\frac{k}{2}, & 1+k-\frac{h}{2}\left(1-u_{E,3}\right), & -\frac{k}{2} & \cdots \\ & \cdots & \cdots & \cdots & \\ 0 & \cdots & 1/3, & -4/3, & 1 \end{array}\right)u^{⋆⋆} & \\ =RHS,\; \end{array}$$ (10)

where *RHS*_1_ = *RHS*_*n*_*seg*__ = 0. Elements *u*_*E*,*j*_ denote the *j*−th elements of the Euler estimate vectors *u*_*E*_. Note that this system has bandwidth 5, not 3. However, the upper and lower 2nd sub/super-diagonals contain only one element (1/3). As LeVeque [17] points out, this only *slightly disturbs the tridiagonal structure*. To be able to still use a tridiagonal solver, we modify Eq (10), by using explicit Heun rule estimates for *u*_3_ and *u*_*n*_*seg*_−2_ and move those to the right hand side: that is,

$$\begin{array}{cc} RHS_{1}\to -\frac{1}{3}u_{3}^{est},\;\text{and} & RHS_{n_{seg}}\to -\frac{1}{3}u_{n_{seg}-2}^{est}, \end{array}$$

and remove the first and *n*_*seg*_-th row elements 1/3 in Eq (10). The *u*_1_ and *u*_*nseg*_ elements can be updated after the tridiagonal system solution to correct for the explicit estimates, if desired. We find no discernible differences doing so.

Three special cases remain: *n*_*seg*_ = 1, 2, 3.

- If *n*_*seg*_ = 1, only the logistics term can be used: set *A*_*y*_ = 0 in (11b) and (11c) and update the single element $u_{1}^{⋆⋆}$. This must be kept non-negative, however.
- Surprisingly, the *n*_*seg*_ = 2 is the most awkward. Eq (11) use the 2nd order differencing operator on the old (previous time step) solutions,
  $$\begin{array}{c} A_{x}=\left(\begin{array}{cc} -2 & 1\\ 1 & -2 \end{array}\right), \end{array}$$
  and likewise *A*_*y*_. The resulting solutions of Eqs (9a) and (9d) contain limited derivative information, hence the Neumann boundary conditions require $u_{1}^{⋆}=u_{2}^{⋆}$, $u_{1}^{⋆⋆}=u_{2}^{⋆⋆}$, and *u*_1_(*t* + *h*) = *u*_2_(*t* + *h*) and all these must be non-negative. Similar restrictions are also made on the Euler estimate in (11b).
- When *n*_*seg*_ = 3 the resulting 3-vector equations for *u*^⋆^ *u*^⋆⋆^, and *u*(*t* + *h*) all take a form
  $$\left(\begin{array}{ccc} 1 & -4/3 & 1/3\\ S_{1} & S_{2} & S_{1}\\ 1/3 & -4/3 & 1 \end{array}\right)\left(\begin{array}{c} u_{1}\\ u_{2}\\ u_{3} \end{array}\right)=\left(\begin{array}{c} 0\\ RHS_{2}\\ 0 \end{array}\right).$$
  A couple of simple manipulations show *u*_1_ = *u*_2_ = *u*_3_, which says that there is no derivative information if Neumann boundary conditions are imposed on this *n*_*seg*_ = 3 case. The solution is *u*_2_ = *RHS*_2_/(2*S*_1_ + *S*_2_), and thus we get the other two. They must also be kept non-negative.

## World-wide hominin dispersal

We now focus on our principal application using the methods presented above: the world-wide dispersal of *Homo sapiens* out of Africa.

### Capacity maps

Our construction of a time-dependent K uses Net Primary Productivity (NPP) as a proxy [1]. The Miami model [20] was originally formulated in 1972 to estimate NPP (in (grams of carbon in dry organic matter)/m^2^/day) from annual temperature and rainfall [21]. In order to compute our NPP maps, we obtained the temperature and precipitation data from simulations by the bridge program [22] organized at the University of Bristol [23–26]. The simulation data that we use are computed on a 96 × 73 grid, which we interpolate to size 720 × 360 and convert to NPP maps [27] by applying the formulas given in [20].

World—wide NPP data are difficult to obtain, so our Miami model-like maps are somewhat rough. Via testing, we found our Godunov solver to be quite robust with respect to abrupt **x**−steps in the carrying capacity K(x,t).

### Time interpolation of maps

We assembled 61 NPP maps, from 120 kya to 1 kya. These are in 4 kilo—year steps for the first 10; 2 ky steps for the next 21; then 1 ky steps for the remainder. The time stepper in our Godunov scheme has no information about continuity between these discrete NPP maps, so an interpolation scheme needs to be used.

If we have available some estimates for the carrying capacities $K_{L}$, $K_{H}$ at times *t*_*L*_, *t*_*H*_, one possible estimate for K(t) at times *t*_*L*_ ≤ *t* ≤ *t*_*H*_ in between is a homotopy,

$$\begin{array}{c} K\left(x,t\right)=K_{L}\left(x\right)\left(1-S\left(t\right)\right)+K_{H}\left(x\right)S\left(t\right), \end{array}$$

where a sigmoid function 0 = *S*(*t*_*L*_) ≤ *S*(*t*) ≤ *S*(*t*_*H*_) = 1 smoothly interpolates between lower and higher time frames. There are many choices available, such as that used in [1]. We use the following variant.

**Fig 2:**
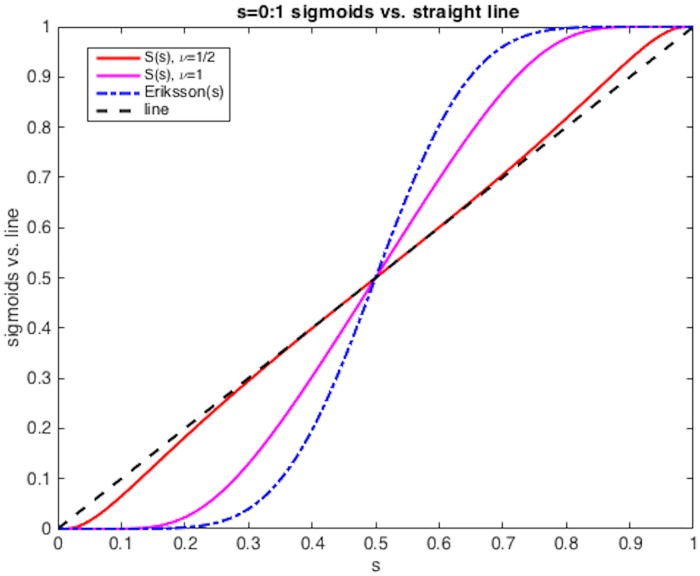
Time frame interpolation methods. The interpolation maps K at different times: sigmoids Eq (12) for *ν* = 1/2 and *ν* = 1, straight line, and Eriksson’s *f*(*f*(*f*(*t*))) model [1]. Time scaling is *s* = (*t* − *t*_*L*_)/(*t*_*H*_ − *t*_*L*_).

Start with the classical sigmoid

$${\begin{array}{c} S\left(z\right)=(1+e^{-z}) \end{array}}^{-1}$$ (11)

which is zero at *z* = −∞ and unity at *z* = +∞. The −∞ < *z* < +∞ interval is not what we want, but the following *t* ↦ *z* transformation permits several variants:

$$z=\frac{2\Delta T\left(t-t_{L}\right)-{\left(\Delta T\right)}^{2}}{{\left(\left(t-t_{L}\right)\left(\Delta T-\left(t-t_{L}\right)\right)\right)}^{\nu }}.$$ (12)

where Δ*T* = *t*_*H*_ − *t*_*L*_. Notice that *S*(*z*(*t*_*L*_)) = 0 and *S*(*z*(*t*_*H*_)) = 1. The exponent *ν* in Eq (12) gives some freedom in choosing a particular form for *z* for almost any *ν* > 0. If *ν* < 1/2, d^2^ *S*/dt^2^ has more than two sign changes, so *ν* ≥ 1/2 is preferable. With choice *ν* = 1/2, the interpolant is nearly a straight line: see Fig 2. However, its turn-up at *t* = *t*_*L*_ and turn-down at *t* = *t*_*H*_ numerically resemble very quick derivative changes. So, we choose *ν* = 1 which also makes *z*(*t*) time scale independent. At both ends, all derivatives of *S*(*z*(*t*)) in *t* smoothly vanish. We also have the forward/backward symmetry *S*(*z*(*t*_*H*_ − *t*)) = 1−*S*(*z*(*t*)) for *t*_*L*_ ≤ *t* ≤ *t*_*H*_, as does [1].

### Parameter optimization

Other tasks remain: optimize the *r* = *λ* and *D* = 2*c* parameters in Eq (1); choose our starting value *T*_*s*_, the start time for our simulation; and choose a threshold value for arrival. For example, a migration wave reaching some fraction of the local carrying capacity at a studied site would constitute *arrival*. Roughly, the distance from Eritrea/Djibouti to any site, arriving at time *t*_*A*_, looks like

$$\begin{array}{c} \text{distance}=a\sqrt{rD}\;t_{A}+b\sqrt{D\;t_{A}} \end{array}$$

where *a*, *b* are unknown fractions of the distance. The terms consist of a nearly constant speed ($∼\sqrt{rD}$) traveling wave, and a diffusion $\sqrt{Dt_{A}}$, respectively. This is a scaling argument using the units *D* ∼ km^2^/yr and *r* ∼ yr^−1^. A fraction ccFRAC≈ 1/2 of the carrying capacity would mostly probe the first term, $a\sqrt{rD}\;t_{A}$. Conversely, a tiny value of ccFRAC weighs too much of the diffusion tail $b\sqrt{Dt_{A}}$. The right-hand frame of Fig 1 shows that the wave consists of a steep traveling wave with extended diffusion tails. We found that arrival times are relatively insensitive to values of ccFRAC ∼0.2. Larger values can produce much later arrival times, as in Table 2 and its missing error bars. Hence, our choice is ccFRAC = 0.1: When the incoming population reaches 10 percent of the local carrying capacity, it has *arrived*. For this ccFRAC choice, the differences between NPP and FEP (*Food Extraction Potential* [28]) arrivals did not seem significant. The opening of the Bering Straight “bridge” occurs before any of our simulation start-times, *T*_*s*_, so arrival times in the Americas become almost linearly increasing for *T*_*s*_ ≥ 45kya. Hence, *T*_*s*_ = 45kya value is a reasonable lower limit. Our NPP data and masks are later than 120 kya, after the beginning of the Tarantian period. A Bering Strait crossing was possible until the Holocene period began around 10 kya. Thus migration data would permit a transit until closure. Arrival in northeastern India is known to have happened before 40kya, so *T*_*s*_ cannot be that small. Thus to limit our optimization space, we choose *T*_*s*_ = 45kya. For this choice of *T*_*s*_, we still have to choose *r*, *D*. Some estimates were known [4, 5] to be approximately *D* ≈ 200km^2^/yr and *r* ≈ 2.0 × 10^−3^ yr^−1^. With the hope that we might be more precise, we optimize on the following RMS parameter,

$$\begin{array}{c} \text{RMS}=\sqrt{\frac{1}{2}\left({\left({\text{sim}}_{MC}-{\text{arch}}_{MC}\right)}^{2}+{\left({\text{sim}}_{FC}-{\text{arch}}_{FC}\right)}^{2}\right)}, \end{array}$$

where arch_*MC*_ = earliest estimated population of Meadowcroft, PA; similarly, arch_*FC*_ = earliest known population of Fell’s Cave, near the tip of Cape Horn, Isla Grande, Tierra del Fuego [29, 30]. Our simulation values sim_*MC*_ and sim_*FC*_ depend on the diffusion/growth parameters *r*, *D*, the *arrival* threshold ccFRAC, and starting time *T*_*s*_.

**Table 2:** Predicted arrival times for 10 sites [29–31]. The furthest apart American sites†, Meadowcroft and Fell’s Cave, were used for optimization. Error estimates give approximate sensitivities to the coarse *r* steps in the left-hand plot of Fig 3. Entry **NA** means the half-filling wave did not arrive. All values are in kilo—years before present.

Early Archaeological sites			
site	arch. range	arrival at 10% CC	arrival at 50% CC
Meadowcroft†	16–14	19.0 ± 0.7	17.5
Taima-Taima	14 ?	13.4 ± 0.9	11.8
El Inga	9	13.2 ± 0.9	11.8
Pachamachay	12–10.5	17.4 ± 0.7	NA
Pikimachay	22.2–12	12.5 ± 0.9	11.4
Pedra Furada	12–5	11.4 ± 0.9	10.3
Tagua-Tagua	11.5	13.4 ± 0.9	11.8
Monte Verde	14.6–12.5	9.9 ± 0.9	8.6
Page-Ladson	14.6	19.4 ± 0.7	18.3
Fell’s Cave†	11	9.3 ± 1.0	7.6

### Population dispersal

**Fig 3:**
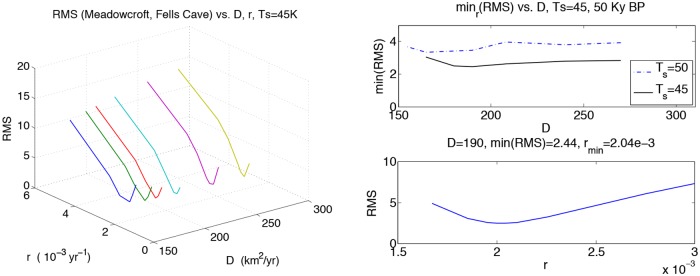
Diffusion and growth parameters optimization. Left: the RMS value of simulation compared to earliest known populations of Meadowcroft and Fell’s Cave. The approximate optimal value of *D* is 190km^2^/yr. The minimum RMS value is shallow in *D* but sensitive to *r* = *λ*, showing we are probing both the traveling wave and diffusion tail. Right: a detailed min_*r*_(*RMS*) optimization in *D* and in *r* at *D* = 190: *λ*_*min*_ = *r*_*min*_ = 2.04 × 10^−3^ yr^−1^.

**Fig 4:**
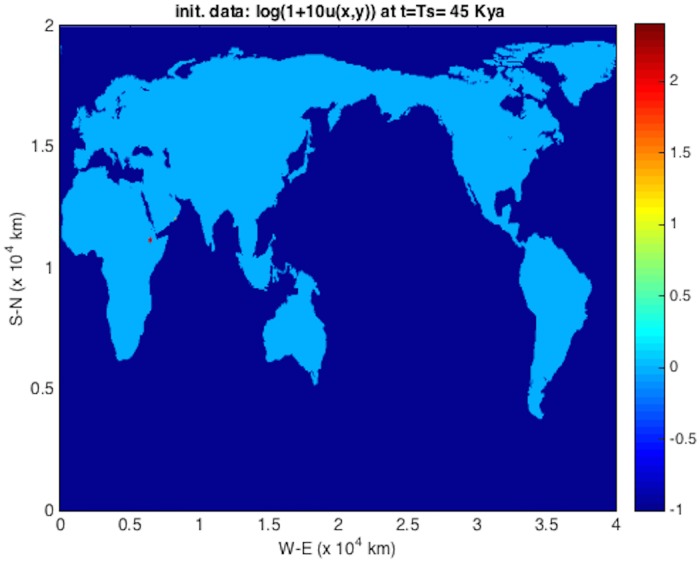
Color-coding and labeling for all of out-of-Africa dispersal plots. Dark blue shows *water*, with K=0. Growth rate is *λ* = 2.04 ⋅ 10^−3^ y^−1^, and diffusion coefficient *D* = 190km^2^/y. The initial distribution at *T*_*s*_ = 45 kya is denoted by a small dark red spot in Eritrea, west of the Bab al Mandab straight: a *σ* = 3 pixel Gaussian with peak *u* = local K=0.72, color scale 2.1.

**Fig 5:**
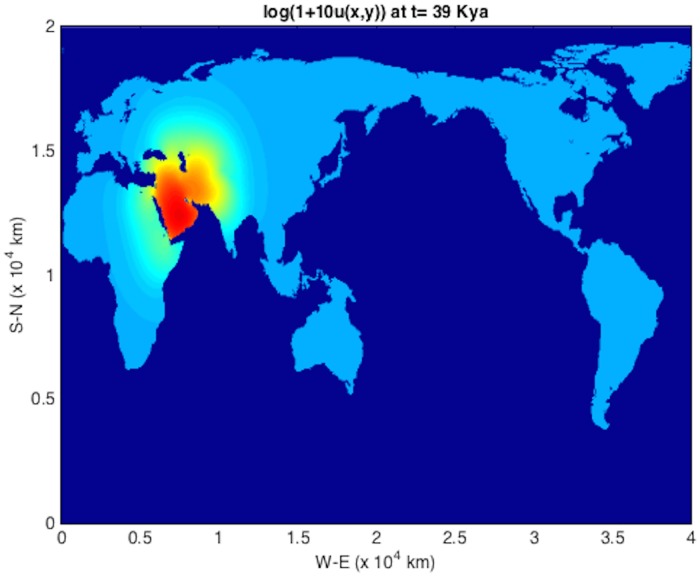
log(1 + 10*u*) plot for out-of-Africa dispersal population distribution at *t* = 39 kya.

**Fig 6:**
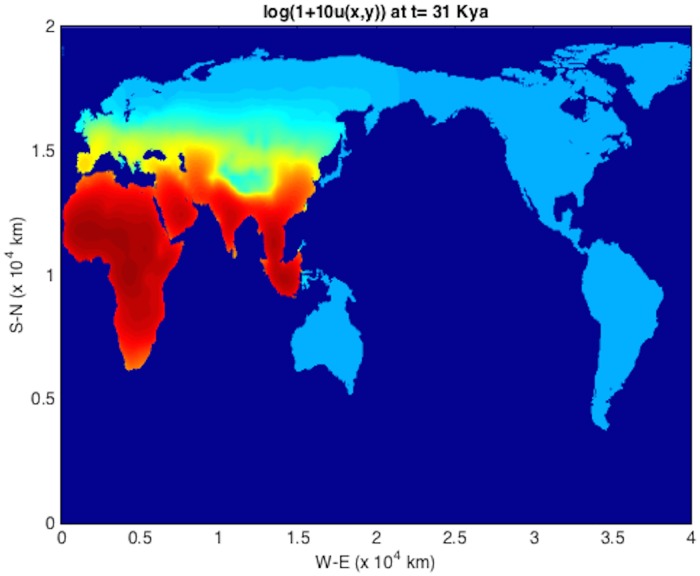
log(1 + 10*u*) plot for out-of-Africa dispersal population distribution at *t* = 31 kya.

**Fig 7:**
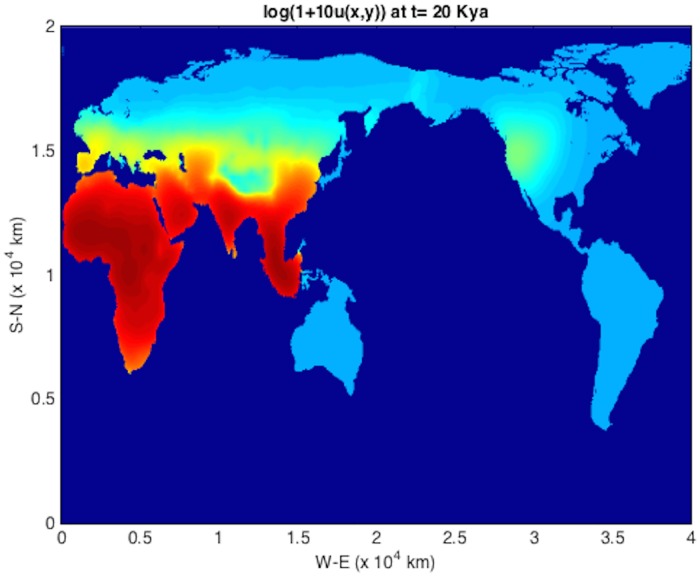
log(1 + 10*u*) plot for out-of-Africa dispersal population distribution at *t* = 20 kya. Notice that the bridge across the Bering Straight is not yet closed.

**Fig 8:**
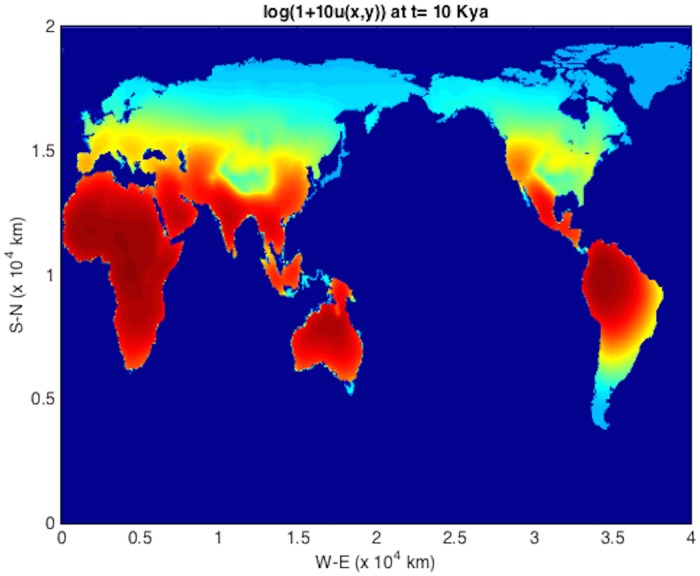
log(1 + 10*u*) plot for out-of-Africa dispersal population distribution at *t* = 10 kya. The bridge across the Bering Straight is now closed.

**Fig 9:**
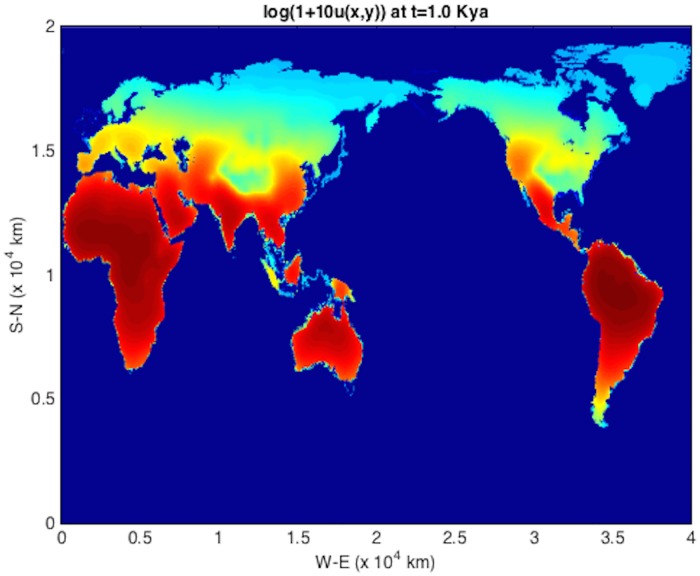
log(1 + 10*u*) plot for out-of-Africa dispersal population distribution at *t* = 1 kya.

An initial population at *t* = *T*_*s*_ = 45 kya at an Eritrean is shown in Fig 4. The integration units are scaled following Table 1 such that *λ* = 2.04 ⋅ 10^−3^ y^−1^ and *D* = 2*c* = 190 km^2^/y (consistent with those used in [4]). The solver is then run using time frame K maps described above, down to 1 kya before present. The remaining plots (Figs 5, 6, 7, 8 and 9) display the resulting population dispersal simulation on space-time dependent K maps. Using population parameters optimized in Subsection **Parameter optimization** gives results consistent with the literature. The gross features of the late (45-50 kya) out-of-Africa dispersal of *Homo sapiens* are reproduced [32], e.g. the colonization of Western Europe before 40 kya and that of South America before 14 kya [31]. As the upper plot in the right-hand panel of Fig 3 shows, for these (*r*, *D*) parameters, arrival times increase as *T*_*s*_ ≥ 45 increases. Figs 4, 5, 6, 7, 8 and 9 show our results graphically. Furthermore, the predictions of arrival times shown in the table of Table 2 are also quite reasonable considering the uncertainties involved in our crude NPP maps. The 2^*nd*^ column of Table 2 also indicates archaeological uncertainties.

## Conclusions

In this paper, we present a semi-implicit Godunov scheme for the Fisher/KPP equation with a variable carrying capacity K. In one dimension, the expected traveling wave [10, 11] develops as shown in Fig 1. As in the one-dimensional situation, a traveling wave also develops as expected [11] in 2-D. Plots in Subsection **Population dispersal** clearly show these evolving waves.

In the Section **Space-time dependent capacity maps** we describe our procedure for handling **x** and time dependent K(x,t), Eq (9).

Finally, we apply our scheme to our principal objective of population dynamics: the out-of-Africa dispersal of *Homo sapiens*. On the Mercator projected world map, by using vegetation net primal productivity (NPP) as a proxy for carrying capacity, we get Table 2 which shows that interpolating in time between discrete K- maps yields stable and reasonable results. These results show ancestor arrival in NE India before 40 kya, then a crossing of the Bering Strait before 10 kya. Multiple routes into South Asia [33] are also evident. Honesty requires that we admit our size (56km)^2^ pixels do not resolve the two crossing points at Bab al Mandab and Sinai adequately. Additionally, we make no attempt to model coastlines effectively. Neither simple boats nor easier passages along beaches are included in our model: only Neumann boundary conditions determine such movements here. Better modeling of coastlines and more detailed NPP maps would improve our results.

The core computations performed by our solver are *independent* tridiagonal solutions, which can be easily parallelized to deal with larger grids (e.g. [34], p. 116). In order to improve numerical performance, in the Appendix **Map segmentation**, we discuss a compressed storage scheme to integrate the Fisher/KPP equation on a projected world map. About 71% of the earth’s surface is water, so this compressed storage reduces computational work by the same amount.

## Provenance

For this paper, the simulations were run on either a Mac Mini, 2.3 GHz Intel Core i7, or a 2.8 GHz i7 MacBook (both OS 10.9.5). On the Mini, MatLab R2015b was used, R2015a on the MacBook laptop. Our codes and maps are available [27].

## Map segmentation appendix

**Fig 10:**
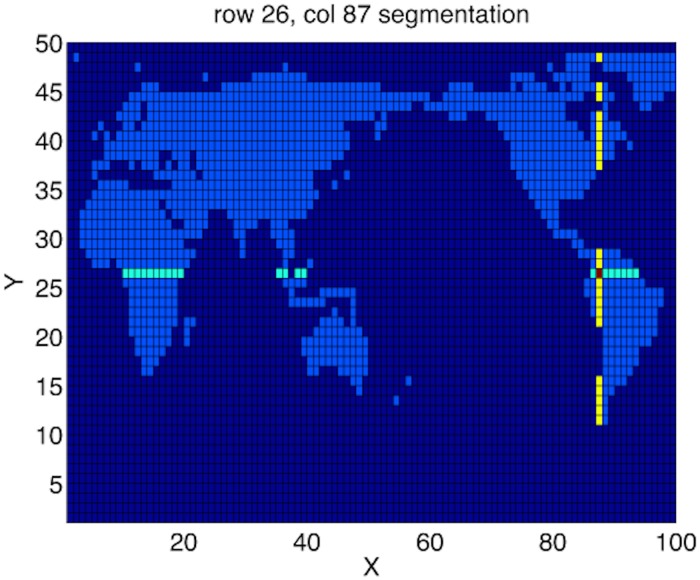
Habitable map regions. Land is set to 1 (light blue) and water 0 (dark blue) on a low-resolution *N*_*x*_ = 100, *N*_*y*_ = 50 grid. Row 26 has 4 habitable segments (cyan), column 87 has 5 segments (yellow).

Our solver on a geographical map uses a map outline, i.e., a rectangular grid with 1’s in habitable regions, and 0’s in the water, see Fig 10. Each Godunov direction step only involves independent rows, resp. columns, indexed by starts/ends of habitable segments. Independent columns, *i* = 1…*NX* in Fig 10 will have nysegs(i) of habitable segments, whose start and end positions are ystart_seg(k) and yend_seg(k), respectively, where *k* = 1…nysegs(i). Likewise, for *j* = 1…*NY* rows, each have nxsegs(j) also with start/end positions. A 100 × 50 example, Fig 10, shows row 26 has 4 segments of varying size. Column 87 has 5 segments.

Each time a new carrying capacity map is loaded (up to 61 total) [1, 22] a suitable mask is computed from $K_{L}$ and $K_{H}$, then resegmentation is performed: see Subsection **Time interpolation of maps**. Resegmentation facilitates time changing coastlines.

We include here an illustrative MatLab code sample of our Godunov-Strang-Yoshida scheme. Notation: cfl = Eq (4), carrying capacities kL at *t*, kH at *t* + *h*. Over multiple time-steps, godunovstep1 which is used in the two half-steps, *h*/2, of Eq (9) can be doubled up: at step *t*_*n*+1_ Eq (9a) can be combined with Eq (9d) used at *t*_*n*_ for one full step, *h*. This is common for all trapezoidal rule integrations: for example, equation (2.1.4) in [35], or in a stochastic setting [36].

% NY X direction updates for 1/2-step1, Y direction for step2

 locx = 0;

 for j = 1:NY

    nsegs = nxsegs(j);

    for k = 1:nsegs

      istart = xstart_seg(locx+k); iend = xend_seg(locx+k);

      ninseg = iend-istart+1;

      u0(1:ninseg) = u(istart:iend,j);

% Eq (9a) solution, sc1, sc2 are static workspaces

      ut = godunovstep1(ninseg,h,cfl,u0,sc1,sc2);

      u(istart:iend,j) = ut(1:ninseg);

    end

    locx = locx + nsegs;

 end

% do NX Y dir. updates step2, wt0,wt1 = time interpolation weights

 locy = 0;

 for i = 1:NX

    nsegs = nysegs(i);

    for k = 1:nsegs

      jstart = ystart_seg(locy+k); jend = yend_seg(locy+k);

      ninseg = jend-jstart+1;

      u1(1:ninseg) = u(i,jstart:jend)';

      kL(1:ninseg) = w_L(i,jstart:jend)*(1-wt0) …

               + w_H(i,jstart:jend)*wt0;

      kH(1:ninseg) = w_L(i,jstart:jend)*(1-wth) …

               + w_H(i,jstart:jend)*wth;

% Eq (9c) solution:

      ut = godunovstep2(ninseg,h,cfl,u1,sc1,sc2,kL,kH);

      u(i,jstart:jend) = ut(1:ninseg);

    end

    locy = locy + nsegs;

 end

% Repeat godunovstep1, as above for Eq (9d)

% locx = 0;

% for j = 1:NY

%    ETC

% end
